# Exploring Ambient Artificial Intelligence to Enhance Learning and Feedback During Operating Room-to-Intensive Care Unit Handoffs: Co-Design and Simulation Study

**DOI:** 10.2196/85666

**Published:** 2026-07-02

**Authors:** Laleh Jalilian, Federico Lorenzo Barra, Tristan Grogan, Jason Lee, Achuta Kadambi

**Affiliations:** 1 Department of Anesthesiology and Perioperative Medicine, University of California, Los Angeles, 757 Westwood Plaza, Suite 3325, Los Angeles, CA, 90095, United States, 1 (310) 267-6629; 2 Department of Education - General Directorate, Azienda Ospedaliero-Universitaria "Maggiore della Carità", Novara, Italy; 3 Department of Medicine Statistics Core, David Geffen School of Medicine, University of California, Los Angeles, 1100 Glendon Avenue, 8th Floor,Los Angeles, CA, 90024, United States; 4 Electrical and Computer Engineering, University of California, Los Angeles, 420 Westwood Plaza,Los Angeles, CA, 90095, United States

**Keywords:** simulation training, handoff communication, human-computer interaction, ambient artificial intelligence, prompt engineering

## Abstract

**Background:**

Operating room (OR)-to-intensive care unit (ICU) handoffs are among the most complex and high-risk communication events in perioperative care. Despite the implementation of structured checklists, trainees often receive limited feedback on their communication skills, and simulation-based education rarely provides objective data on communication performance and checklist adherence. This study explores how an ambient artificial intelligence (AI) handoff assistant used during simulation-based training of OR-to-ICU handoff discussions can enhance clinical communication training and AI literacy by mapping spoken handoff discussions to handoff checklist items, providing immediate feedback on checklist item omissions, and generating a structured handoff note that functions as a feedback-rich learning artifact.

**Objective:**

This study aims to co-design and evaluate an ambient AI handoff assistant that transcribes spoken OR-to-ICU handoff communication, maps the discussion to handoff checklist items, generates a structured handoff note for educational review, and provides immediate feedback on handoff completeness during simulated OR-to-ICU handoff discussions in a low-fidelity educational setting.

**Methods:**

A 2-phase mixed-methods study was conducted within the University of California, Los Angeles, Department of Anesthesiology and Perioperative Care (July-October 2025). Phase 1 comprised co-design interviews with 4 clinician educators to identify limitations of current handoff training and inform AI feature development. Phase 2 involved an error analysis, as well as evaluations of usability, workload, and educational impact, conducted through ten 60-minute simulation sessions with pairs of medical students and first-year residents. Quantitative measures included the Physician Task Load Index, System Usability Scale, and a postsimulation survey; qualitative data from co-design sessions and simulation debrief interviews were thematically analyzed.

**Results:**

Educators highlighted inconsistent checklist use and the absence of objective feedback on learners’ communication skills as key areas that could benefit from structured documentation of handoff discussions using AI. Error analysis of the ambient AI handoff assistant revealed a mean of 3.6 (SD 1.2) errors per note, with incorrect output being the most frequent error type. There was no statistically significant difference between the ambient AI handoff assistant and the paper checklist with respect to the Physician Task Load Index and System Usability Scale measures. Trainees valued real-time transcripts and structured handoff notes for reflection of communication practices, and exposure to AI documentation errors enhanced critical thinking and awareness of AI technology limitations.

**Conclusions:**

The ambient AI handoff assistant mapped simulated handoff discussions to checklist items and generated a structured handoff note, facilitating reflection on team-based communication skills in handoff education. Imperfections in the AI’s output encouraged critical appraisal of its capabilities and prompted discussion about automation complacency, suggesting that AI-assisted simulations can foster both communication and digital literacy skills essential for future AI-enabled clinical practice.

## Introduction

Effective handoff and transitions of care discussions are essential for ensuring safe and high-quality patient care [1-4]. Operating room (OR)-to-intensive care unit (ICU) handoffs are particularly complex and high risk, requiring coordinated communication among surgical, anesthesia, and ICU teams under conditions of cognitive load, time pressure, and frequent interruptions [5-7]. Incomplete communication transfer during OR-to-ICU handoffs remains a preventable contributor to medical errors and adverse patient outcomes [6,8,9], and structured handoff checklists have been developed to standardize communication and improve information transfer [10-15]. Despite the introduction of checklists, adherence to checklists remains variable [16], and trainees often receive limited instruction or feedback on communication skills [17]. Handoff skills are frequently learned informally through observation, with variable role modeling, inconsistent feedback, and few opportunities for deliberate practice [18]. As a result, trainees may be unaware of omissions or deficiencies in their communication and may not learn important relational communication skills. Without objective assessment, gaps between knowledge and performance may persist, contributing to preventable harm. Simulation-based education offers a safe environment to practice handoffs, but most simulations rely on subjective observation. Learners may overestimate the completeness of their handoffs, and without objective records or data-driven feedback, meaningful self-assessment and improvement are limited [19].

Recent advances in ambient artificial intelligence (AI) present new opportunities to address this educational gap. Ambient AI systems use natural language processing and large language models (LLMs) to passively capture spoken communication and generate summarized documentation of the transcript. Applied to checklist-based handoffs, such systems are being explored for their potential to identify which checklist items are verbalized, map spoken communication to structured checklist elements [20], and provide feedback on the completeness of information exchange. This capability may enhance handoff education from subjective assessment to measurable performance analytics, enabling iterative practice and self-reflection. Despite growing interest in AI applications for documentation and workflow support, no prior studies have examined the use of ambient AI for enhancing handoff communication skills to medical trainees [21,22]. This gap is particularly striking given that standardized handoff checklists already provide structured frameworks that align well with LLM-based mapping through prompt engineering, and simulation-based handoff training is widely implemented yet lacks robust, objective assessment tools [23] of verbal discussions [24].

To address this need, we developed and evaluated an ambient AI-assisted handoff simulation platform designed to support learning and feedback during OR-to-ICU handoff educational sessions. The system listens to trainee handoff discussions, maps verbalized content to checklist items, and generates structured documentation identifying which items were omitted. The objectives of this study were to (1) identify and co-design features of an ambient AI handoff assistant for OR-to-ICU handoff education through needs assessment and interviews with clinician educators, focusing on limitations of current handoff training and design requirements for AI-mediated feedback, (2) assess the performance and accuracy of the LLM-based checklist validation in identifying discussed and omitted handoff items, and (3) evaluate the usability, workload, and learning impact of the AI-assisted handoff simulation platform during low-fidelity sessions, while exploring learner perceptions of the AI-generated feedback, specifically its influence on communication, reflection, and perceived educational and clinical value.

## Methods

### Overview

A 2-phase, mixed-methods study was conducted within the University of California, Los Angeles (UCLA), Department of Anesthesiology and Perioperative Care (July-October 2025). The COREQ (Consolidated Criteria for Reporting Qualitative Research) [25] (Checklist 1) framework was used to guide research reporting.

### Study Design

We used a human-centered, co-design methodology to develop an ambient AI handoff assistant. Co-design was selected for its suitability in simulation-based health care research, as it enables clinicians to surface workflow challenges and limitations, shape system features, and envision technology integration in high-stakes team communication [26,27]. The study was conducted in two phases: (1) needs assessment and co-design interviews with OR-to-ICU quality improvement leaders, aiming to (i) identify limitations of the current handoff educational modules, (ii) elicit expectations and perceptions of automated handoff discussion capture using an ambient AI handoff assistant, and (iii) prioritize design features for the ambient AI handoff assistant for handoff education; and (2) low-fidelity simulation sessions to evaluate the educational use of the ambient AI handoff assistant, with mixed-methods assessment of experience, acceptance, usability, and workload.

### Participants and Setting

For the Needs Assessment and Co-Design workshop, 4 clinician educators participated in semistructured 45-minute interviews (remote or in person). Their years of professional experience ranged from 9 to 24, with a mean of 16 (SD 4.9) years. The decision to interview educators individually was dictated by their availability to meet with the study team. In the simulation phase, 10 simulation sessions were conducted in a low-fidelity setting. Each session involved 2 medical students or first-year interns who had no prior exposure to the OR-to-ICU handoff process at UCLA, who played the roles of surgery and anesthesia, and played the role of the ICU attending. New participants were recruited for each simulation session to minimize bias from repeated exposure. Demographic breakdown of simulation participants is listed in Table 1.

**Table 1.** Overview of simulation study participants (n=20).

Participant type	Number of participants
What is your current training level?	
Medical student	7
Resident	13
Sex	
Male	7
Female	10
Nonbinary	2
Prefer not to answer	1
Do you have experience using an ambient AI^a^ scribe? (An AI scribe refers to a tool that automatically generates documentation from ambient audio or speech input during clinical encounters, such as DAX Copilot or Nabla.)	
Yes	2
No	18
Do you have experience using generative AI technologies? (Examples include ChatGPT, Claude, Med-PaLM, Microsoft Copilot, or other tools that use artificial intelligence to generate content, summarize information, or support documentation.)	
No, I have never used any AI-generated technologies.	1
Yes, I’ve tried one briefly out of curiosity.	3
Yes, I’ve used them occasionally for general tasks (eg, answering questions, drafting emails).	9
Yes, I’ve used them occasionally for clinical or work-related tasks (eg, summarizing notes, generating documentation).	1
Yes, I use them frequently and consider them an integral part of my workflow.	6

a AI: artificial intelligence.

### Needs Assessment and Co-Design Interviews

The primary study author interviewed 4 clinician educators in a 45-minute semistructured interview fashion using the Clinician Educator Interview guide in Multimedia Appendix 1 after obtaining verbal consent. The interviewer was a clinician trained in the use of qualitative research techniques. Given the interviewer’s role as a faculty peer, reflexivity was emphasized to reduce potential bias. Interviews began with reflections on recent OR-to-ICU handoffs and handoff education to ground discussions in real clinical practice. Clinician educators identified limitations of current handoff educational methods and potential roles for ambient AI in encouraging educational discussion on checklist completeness and communication quality. The interviewer then introduced a design probe, an ambient AI handoff assistant to be used during handoff education simulation sessions with junior trainees. A functional prototype was used to ground discussions in concrete interactions. Using whiteboards/Miro and the ambient AI handoff assistant prototype, participants sketched desired AI functions (eg, transcription verification, structured note review, and interaction styles) and collaboratively prototyped ideal AI-supported simulation workflows. Transcripts were member-checked and coded in Atlas.ti (ATLAS.ti Scientific Software Development GmbH) using a grounded theory approach until thematic saturation was reached.

### Ambient AI Handoff Assistant Technology Development

**Figure 1.**
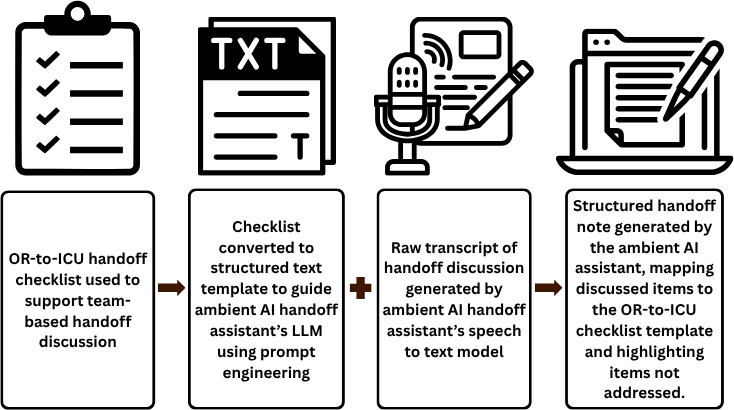
Standardized handoff checklists, such as the University of California Los Angeles operating room-to-intensive care unit Handoff Checklist, can be converted into structured text templates to guide the ambient artificial intelligence handoff assistant in generating documentation aligned with the desired format. Figure adapted from Jalilian et al [28]. AI: artificial intelligence; ICU: intensive care unit; LLM: large language model; OR: operating room.

Based on insights from the needs assessment and co-design interviews, we developed an ambient AI handoff assistant to support both clinician educators and learners during OR-to-ICU handoff simulation sessions. The assistant digitally displays the UCLA OR-to-ICU Handoff Checklist, provides real-time visual transcription of the clinical discussion, and automatically generates a structured handoff note where the discussion is mapped to individual checklist items for learner review. It also highlights checklist items that were not discussed, helping educators and learners identify omissions and communication gaps. A commercial ambient AI scribe was not used because existing systems lack the capability to map spoken handoff dialogue directly to a structured handoff checklist for the purpose of assessing checklist adherence. Meeting this requirement necessitated custom prompt engineering of an LLM aligned specifically to UCLA’s OR-to-ICU handoff checklist. Figure 1 illustrates the technical framework used to map handoff discussion content to corresponding checklist items.

**Figure 2.**
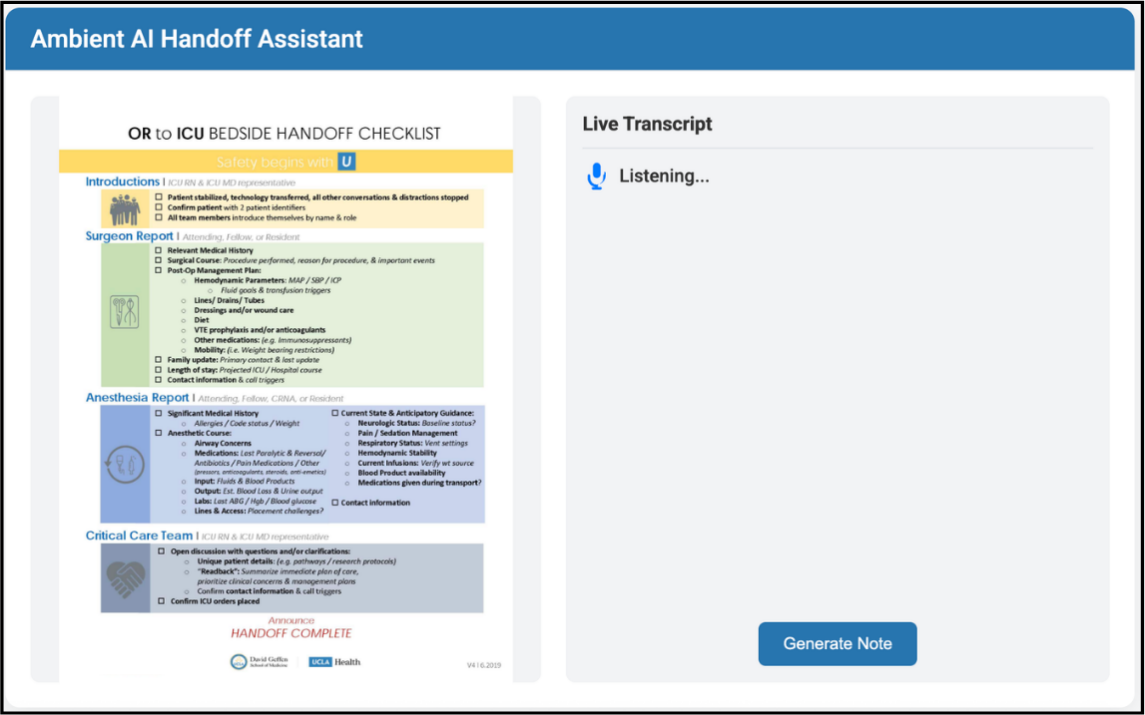
Screenshot of the ambient artificial intelligence handoff assistant pairing digital checklist display with visualization of real-time transcription. The University of California Los Angeles operating room-to-intensive care unit handoff checklist is digitally displayed as a visual cognitive aid for learners, who can simultaneously view a real-time transcription of the discussion in the right-hand panel. AI: artificial intelligence; ICU: intensive care unit; OR: operating room.

**Figure 3.**
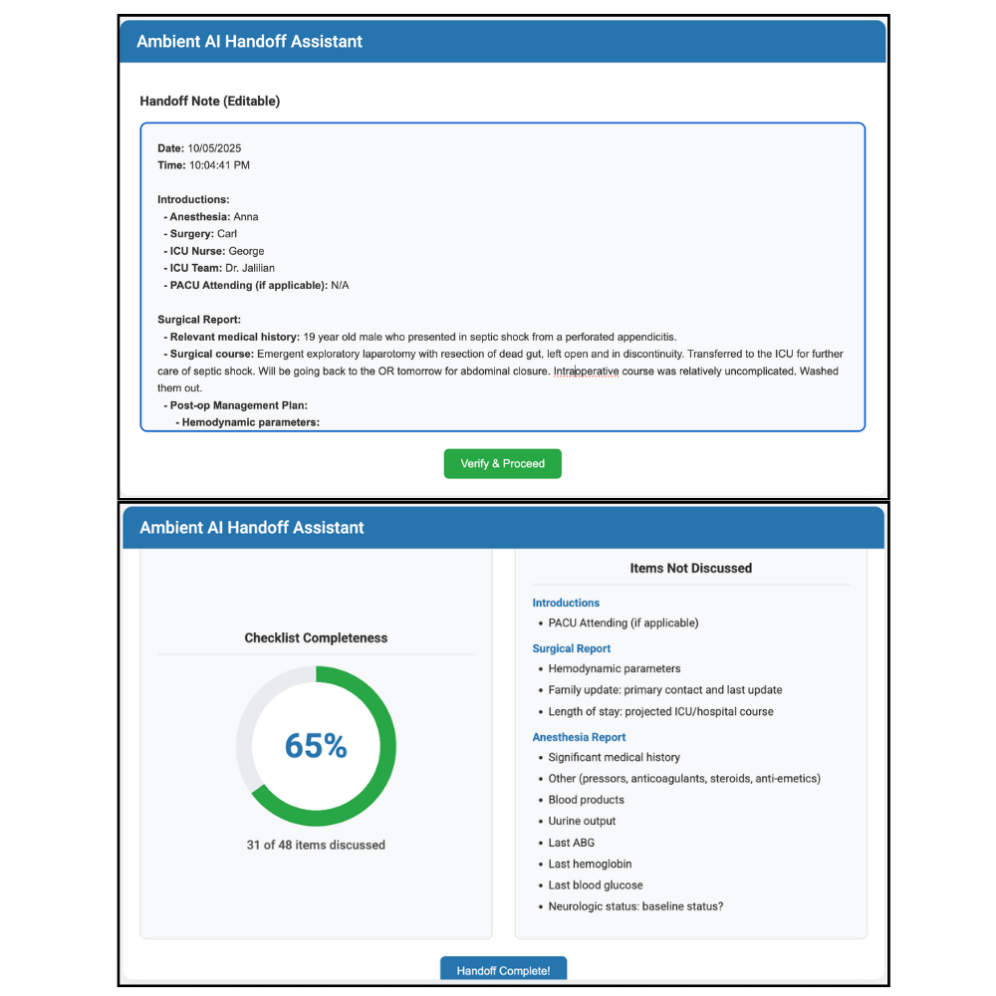
Screenshot of the ambient artificial intelligence handoff assistant as a supportive tool for checklist omission detection. In the top panel, handoff participants view an editable note organized in a checklist-based format, in which discussed content is mapped to its corresponding checklist items, facilitating post-hoc identification of omissions compared with a traditional narrative format. In the bottom panel, items flagged by the artificial intelligence Handoff Assistant as “not discussed” are displayed at the end of the session, providing explicit automated feedback to further support gap identification. Both the checklist-based note in the top panel and the AI-flagged omissions in the bottom panel operate after the discussion has concluded, serving complementary roles by enabling structured human review while also providing explicit, automated feedback to reinforce omission identification. AI: artificial intelligence; ICU: intensive care unit; OR: operating room.

Google AI Studio was used to develop the ambient AI handoff assistant, a single-page application built with HTML5, CSS3, and TypeScript, running entirely within the client’s web browser to ensure low latency and data privacy. Real-time audio capture and transcription are handled by the Gemini 2.5 Flash Live application programming interface (API), configured for continuous listening to capture the full clinical dialogue. The captured transcript is processed by the Gemini 2.5 Flash LLM via the @google/genai SDK. No fine-tuning of the model was performed; instead, a zero-shot prompt engineering approach was used. The prompt requirements and handoff template were specified by clinical domain experts to align with UCLA’s OR-to-ICU handoff checklist and are listed in Multimedia Appendix 2. The prompt uses explicit “Chain of Thought” instructions, requiring the model to extract factual data, resolve conflicting information by prioritizing the most recent statement, and strictly mark missing fields as “N/A.” To ensure structured and reliable data extraction, the system leverages the model’s JSON mode with a strict responseSchema, forcing the output into a predictable JSON object containing a formatted OR-to-ICU handoff note. The organization of transcription content into the final checklist structure and the identification of items labeled “not discussed” are performed via deterministic post-processing logic in TypeScript, rather than a separate predictive model. This logic parses the AI-generated JSON, compares it against a master checklist of 38 clinical items, and flags any item as ’missed’ if the value is empty or remains “N/A.” The application’s core logic is managed by a finite state machine that guides the user through distinct phases: listening, processing, verifying, and summarizing. Auditory feedback and status prompts are delivered via the Gemini 2.5 Flash Live API’s SpeechSynthesis interface, with a preselected “en-GB” female voice to ensure a consistent user experience. Screenshots of the different interface views of the ambient AI Handoff Assistant are visible in Figure 2 and Figure 3.

### Error Analysis of the AI Handoff Assistant

Five simulated OR-to-ICU handoff scenarios were developed using a previously established agentic framework [29] to evaluate the ambient AI handoff assistant in generating handoff notes and displaying undiscussed items. The scenarios represented a range of surgical cases commonly encountered in the ICU. Two clinician educators (LJ and JL) simulated the surgeon and anesthesiologist roles by reading the scenarios aloud while the ambient AI handoff assistant operated in the background. After each session, the clinician educators reviewed the AI-generated handoff note for errors. Each identified error was categorized into one of the following error types: omission, irrelevant or misplaced text, wrong output, or addition. This categorization was coded independently by 2 clinician educators (LJ and JL), and disagreements were discussed until consensus was reached.

### Simulation Testing of the Ambient AI Handoff Assistant

Ten 60-minute simulation sessions in a nonclinical, low-fidelity setting were conducted with pairs of medical students or first-year resident physicians who played the roles of anesthesiology and surgery team members during an OR-to-ICU handoff. Participants were recruited through voluntary email invitations and had no academic or evaluative relationship with the study faculty. Demographic characteristics are presented in Table 1. Each simulation was facilitated by faculty experts in handoff communication who held no supervisory role over participants. Reflexivity was emphasized to mitigate potential bias. Two distinct simulation scenarios (A and B) were generated from a previously developed agentic workflow [29]. The study used a within-team crossover design in which each dyad completed two simulated handoffs: (1) Control: a standard verbal handoff using the institutional paper checklist, and (2) Intervention: a handoff supported by the ambient AI handoff assistant. Each team served as its own control to reduce variability in communication skills, teamwork, and experience. Condition order (AI-first vs. control-first) and case scenario (A vs B) were randomly assigned and counterbalanced to control for order and case effects. Following both simulations, participants completed a postsimulation survey and a semistructured debriefing interview (Multimedia Appendix 3). Debriefing interviews were transcribed and returned to participants for member checking. All participants provided verbal informed consent.

### Statistical Analysis

For the needs assessment and co-design phase, inductive content analysis was used to synthesize design insights. Two researchers (LJ and JL) independently coded transcripts, sketches, and field notes to identify (1) limitations in current handoff education and (2) desired ambient AI handoff assistant features. Coding discrepancies were resolved by consensus, and themes were consolidated into preliminary design requirements for the handoff AI assistant. The Educator Co-design codebook is listed as Codebook A in Multimedia Appendix 4.

Quantitative data from the 4-item Physician Task Load Index (PTL) [30] and System Usability Scale (SUS) were analyzed descriptively. Each participant completed both the control (paper checklist) and intervention (ambient AI assistant) conditions in a within-subjects crossover design. For each measure, paired-samples analyses were conducted to compare participant scores between conditions. The normality of paired differences was evaluated using the Shapiro–Wilk test. When assumptions of normality were satisfied, paired *t*-tests were used to compare mean scores between the control and AI conditions. Results are reported as means (standard deviations) for normally distributed data. All estimates include 95% CIs for the mean differences. Effect sizes for paired 2-tailed *t* tests were expressed as Cohen *d*ₙ (standardized mean difference for paired data). Although 2 participants worked together in each of 10 simulation sessions, the paired 2-tailed *t* test was selected as the primary analytic approach because each participant served as their own control, and between-team clustering was minimal. All analyses were conducted using Python (version 3.11; Python Software Foundation), with statistical significance set at *P*<.05 (2-tailed).

For the simulation debriefings, qualitative data from debriefings were coded independently by two researchers (LJ and JL) using Atlas.ti, with themes refined through iterative consensus. Structured observations were analyzed to capture communication dynamics and were triangulated with interview findings to assess human-technology interaction. The Learner Simulation Codebook is listed as Codebook B in Multimedia Appendix 4.

### Ethical Considerations

This study was approved by the UCLA Institutional Review Board #25‐1998. Verbal informed consent was obtained from all participants before data collection. Participants were informed of the study purpose, procedures, potential risks and benefits, and their right to decline participation or withdraw at any time. Measures were taken to protect participant privacy and maintain confidentiality, including deidentification of data and secure storage of study materials.

## Results

### Needs Assessment and Co-Design Thematic Analysis

Themes from the co-design interviews with educators are listed in Table 2. Educators described ongoing challenges in teaching closed-loop communication and ensuring complete information transfer during handoffs. The OR-to-ICU checklist was viewed as a valuable cognitive aid for structuring handoff discussions across surgical, anesthetic, and ICU domains, yet there was recognition that its real-world use was inconsistent and that trainees could inadvertently skip over checklist items. Educators emphasized the need for greater situational awareness and systematic progression through each checklist item during real-time communication.

**Table 2.** Thematic analysis of needs assessment and co-design sessions on ambient AI handoff assistants for operating room-to-intensive care unit handoff education. Representative quotes are drawn from clinician educator interviews conducted during the co-design phase (n=4).

Theme	Key insight	Representative quote
Promoting professional communication	Awareness of AI^a^ documentation may encourage more mindful, deliberate, and professional communication among trainees.	*“I’d be more careful of what I’m saying.”*
Enhancing reflective learning	AI-generated transcripts and note summaries may support self-assessment and reflection, helping learners review and improve their communication.	*“Having a transcript to go back and refer to adds another level of feedback.”*
Exposure and readiness for AI	Exposure in simulation may allow trainees to experience AI-assisted documentation and assess its accuracy and use in realistic settings.	*“Ambient AI scribes might be one of the first AI technologies that perioperative doctors might be exposed to. Would be interesting to see how accurate the handoff assistant is in documenting the discussion.”*
Calibration of trust in AI output for learners	EAmbient AI Handoff Assistant may be more inaccurate than commercial ambient AI scribes, but exposure to AI transcription inaccuracies may enable trust calibration in AI.	*“[Learners] need to see the AI make mistakes. It doesn’t matter how accurate [the ambient AI handoff assistant] is, because exposure to the errors is instructive in and of itself.”*
Reducing documentation burden in simulation environments	Automating note-taking and checklist reconciliation may allow educators to focus on observation and higher-level feedback rather than manual documentation during teaching.	*“It’d be nice to focus on the students instead of taking notes.”*
Ethical and privacy considerations in training	Educators emphasize the ethical obligation to protect learners’ privacy, psychological safety, and autonomy when using AI in training.	*“If they know they’re being recorded, they might not ask questions they think will sound dumb.”*
Trust and validation of AI output for educators	Educators are uncertain of the accuracy of AI-generated content and need experience to understand its educational use.	*“If it can’t tell which handoff checklist items the trainee did or did not mention, I wouldn’t rely on it. It might not be the point of a tool like this though.”*
Design requirements for ambient AI handoff assistant	Digital checklist display, real-time display of transcription, mapping transcript content to checklist categories in the format of a structured checklist-aligned handoff note, and explicitly labeling checklist items as “discussed” or “not discussed” based on transcript evidence would support clinician educators.	*“I don’t want to bring a paper checklist to one of these sessions, so we should digitally display it as part of the module.”*

a AI: artificial intelligence.

We’ve taught the residents to use the checklist for years, but in the heat of the moment, they skip parts of the checklist.

[Clinician Educator]

Educators felt that many trainees struggled with the interpersonal dimensions of team communication, such as speaking hesitantly, not introducing themselves, or not making eye contact with other members of the handoff team. Educators highlighted that in high-stakes transitions of care, clarity, confidence, and engagement are essential safety and communication behaviors that promote shared understanding. Developing these expressive and relational communication skills was seen as critical yet under-taught.

We don’t teach the residents how to speak in a handoff. We focus on what checklist items need to be discussed, not so much on delivery, tone, or engagement with the team.

[Clinician Educator]

Traditional feedback was described as subjective and infrequent, offering limited improvement, especially if an attending physician does not accompany a trainee to the ICU for a handoff.

Without a record of what was actually said, feedback is just opinion. Everyone remembers the handoff a little bit differently.

[Clinician Educator]

Educators perceived AI-generated documentation as a promising way of creating an objective record of what was actually communicated during handoffs. In developing the ambient AI handoff assistant, they noted that AI-assisted documentation technologies could more concretely identify communication omissions and translate them into specific, actionable learning points, while maintaining a safe environment for learners to critically evaluate the limitations of AI.

My understanding is that AI output isn’t perfect, but I guess that would be part of the learning. Trainees would see how the AI interprets them. Maybe it might make them question it or think critically about accuracy.

[Clinician Educator]

Educators felt that the design requirements of the ambient AI handoff assistant should include digital display of the checklist, real-time visualization of transcription, and an AI-generated handoff note where each checklist item is populated with what the learners discussed to enable educator validation of checklist completeness. Beyond the educational module, they suggested that commercially available ambient AI scribe technologies could potentially enhance handoff processes in the future through the generation of a handoff note for the receiving ICU team to use as a reference of the discussion. Introducing ambient AI first in a simulated setting would allow educators and Handoff QI leaders to evaluate their use, feasibility, and user acceptance in simulated handoff workflows before considering any potential real-world use.

### Error Analysis of the Ambient AI Handoff Assistant

There were 18 errors noted in 5 of 5 draft notes (average of 3.6 errors per draft note). Error type by product is detailed in Table 2, with “wrong output” being the most frequent error across products.

### Physician Task Load and System Usability Score

The paired-samples 2-tailed *t* test showed no significant difference in perceived task load between the paper checklist (mean 12.9, SD 16) and the AI handoff assistant (mean 15.8, SD 8.3), a difference of 2.9 (95% CI −5.9 to 11.7; *P*=.50). This suggests that the AI tool did not meaningfully change participants’ subjective workload during simulated handoffs. Participants rated the AI assistant and paper checklist as similarly usable, with mean SUS scores of 81.4 (SD 11.3) for the paper checklist and 74.3 (SD 13) for the AI assistant, a difference of 2.9 (95% CI −0.6 to 14.8; *P*=.06). Although the AI assistant trended slightly lower in usability ratings, this difference was not quite statistically significant (Tables3  4).

**Table 3.** Physician task load and System Usability Scale (n=20).

Metric	Paper checklist, mean (SD)	Ambient AI^c^ handoff assistant, mean (SD)	*t* test (df)	*P* value	Cohen *d*
PTL^a^	12.9 (16)	15.8 (8.3)	0.68 (19)	.50	0.15
SUS^b^	81.4 (11.3)	74.3 (13.0)	−1.93 (19)	.06	−0.43

a PTL: Physician Task Load Index.

b SUS: System Usability Scale.

c AI: artificial intelligence.

**Table 4.** Postsimulation survey results^a^ (n=20).

Postsimulation survey results	Mean (SD)	95% CI
The AI^b^-generated handoff note and missed-items list helped me recognize strengths and weaknesses in my handoff communication.	3.9 (0.9)	3.5-4.3
The AI assistant’s feedback complemented faculty debriefing and peer discussion.	4.3 (0.6)	4-4.6
I was able to distinguish between accurate and inaccurate information in the AI-generated documentation.	3.8 (1)	3.2-4.3
I exercised appropriate caution in interpreting and relying on AI-generated documentation.	3.9 (0.9)	3.5-4.3
Reviewing the AI-generated note and missed-items list increased my confidence in performing OR^c^-to-ICU^d^ handoffs.	4 (1)	3.5-4.4
The AI feedback helped me feel more prepared to apply structured handoff practices in clinical settings.	4.2 (0.9)	3.8-4.6

a Response options of strongly disagree (1), disagree (2), neither agree nor disagree (3), agree (4), and strongly agree (5)

b AI: artificial intelligence.

c OR: operating room.

d ICU: intensive care unit.

A post-hoc power analysis was performed to estimate the minimum detectable effect size under our study’s sample size of 20 paired observations. Assuming a 2-sided *α*=.05, this sample provides 80% power to detect a standardized mean difference of 0.66. In contrast, the observed effect sizes in our data were 0.43 for the SUS outcome and –0.15 for the task load outcome, which correspond to smaller magnitudes than this detectable threshold. To achieve 80% power to detect an effect size of 0.43, a sample size of approximately 45 would be required, whereas detecting an effect as small as 0.15 would require roughly 351 participants. These estimates may guide the design of future studies with adequate power to detect more modest effects.

### Simulation Debrief Thematic Analysis

Themes from debrief interviews with trainees are captured in Table 5. Trainees emphasized the educational value of an ambient AI handoff assistant in simulated handoff training. AI-generated transcripts and structured notes were felt to provide objective feedback, allowing learners to identify omissions, reflect on their communication, and discuss the limits of AI. These outputs transformed team discussions into concrete learning records that supported debriefing and self-assessment, addressing a gap in handoff education often reliant on subjective recall (Table 6).

**Table 5.** Frequency counts, percentages, definitions, and examples of error types in the artificial intelligence-generated operating room-to-intensive care unit handoff note (n=5).

Error type	Ambient AI^a^ handoff assistant, n (%)	Definition	Example
Omission	5 (27.7)	AI assistant leaves out key information that was discussed	*“No mention of estimated blood loss when it was discussed”*
Misplaced text	3 (16.7)	AI assistant output is technically correct but not in appropriate checklist item	*“Estimated blood loss was discussed but placed in the wrong checklist item”*
Wrong output	7 (38.9)	AI assistant provides an incorrect response	*“Phenylephrine was the spoken vasopressor medication but ephedrine was documented instead”*
Addition	3 (16.7)	AI assistant adds inappropriate or irrelevant information	*“Estimated blood loss was described only as ‘minimal,’ but the AI output documents it as ‘minimal (~50 mL).’*
Total	18 (100)		

a AI: artificial intelligence.

**Table 6.** Thematic analysis of simulation debrief. Representative quotes are drawn from learner participants conducted during the simulation phase (n=20).

Theme	Key insight	Representative quote
Exposure and readiness for AI^a^	Learners viewed the simulation as a valuable first exposure to AI tools in clinical communication, where they could evaluate AI capabilities and accuracy.	*“This was my first time using something like this. I was surprised at how well it actually captured what I said.”*
Reflective and professional learning	Reviewing AI-generated transcripts encouraged self-assessment and helped learners recognize patterns in their communication and teamwork.	*“Seeing the transcript was good. I didn’t realize I skipped over things when I talked through a case.”*
Feedback integration and educational use	Learners discuss how they or faculty could use AI transcripts to guide debriefing, identify missed information, or support feedback loops.	*“I liked the feature that highlighted what we didn’t discuss.”*
Communication awareness	Awareness of being recorded made learners more deliberate in how they presented information during handoffs.	*“Knowing it was recording made me think about what I was saying.”*
Peer dynamics and team communication	Learners sometimes adjust how they communicate with teammates when AI is present.	*“We were more formal because we knew the AI was picking up everything.”*
Trust, accuracy, and error sensitivity	Learners noted AI’s limitations and inaccuracies in speech-to-text transcription and note generation, emphasizing the need for human verification.	*“It made mistakes! It was interesting to see how some of the medical language was mistakenly transcribed, like “mcgs” being transcribed as “Mike’s.” I think you will need to check it.”*
Translation to real-world clinical practice	Learners connected the simulation to future practice, envisioning how AI scribes could improve communication and documentation in real OR^b^-to-ICU^c^ handoff workflows.	*“If this worked in the real ICU, the generated note would keep everyone on the same page and be really helpful to refer back to.”*
Emotional and psychological responses to AI	Learners sometimes felt self-conscious knowing AI is “listening.”	*“At first it felt weird knowing it was recording everything. I got used to it though.”*
Ethical and privacy awareness in training	Learners reflected about consent, data storage, and fairness in educational AI use.	*“I’d want to know who can access the data from these sessions.”*

a AI: artificial intelligence.

b OR: operating room.

c ICU: intensive care unit.

Seeing the AI transcript right after the handoff was great. It was interesting to see what I actually said versus what I thought I said.

[Learner]

Exposure to the ambient AI handoff assistant’s errors also promoted critical thinking and awareness of the technology’s limitations.

Seeing the mistakes the AI made in its transcription was helpful. We can’t just go on auto-pilot with it.

[Learner]

Trainees further envisioned how AI scribe technology might impact real-world OR-to-ICU workflows to document complex handoffs, enhance continuity, and strengthen accountability.

If it could document the handoff automatically, it would help with continuity, especially for the ICU team coming on shift.

[Learner]

They did express reservations regarding data governance of ambient AI technologies.

If AI gets implemented in the real-world, it should be clear what data is actually being stored.

[Learner]

## Discussion

### Principal Findings

This study designed and evaluated an ambient AI handoff assistant for documenting OR-to-ICU handoff discussions within a medical education context. Because OR-to-ICU transitions are among the most complex and high-risk discussions in perioperative care, they provided an ideal setting for exploring how ambient AI can support medical trainees in promoting awareness of their communication and checklist completion during high-stakes discussions. Introducing the ambient AI handoff assistant to transcribe participants’ discussions appeared to enhance perceptions of learning and educational value, as reflected in both survey feedback and debrief interviews. Further, PTL scores did not differ significantly between the AI scribe and the traditional checklist. This may suggest that verbally delivering a complex OR-to-ICU handoff is inherently challenging for inexperienced trainees, regardless of whether they are supported by a paper checklist or the AI-based handoff assistant. SUS scores did not differ significantly between the paper checklist and the AI-based handoff assistant, suggesting that digitally displayed checklists may be acceptable to users as a cognitive aid. Participants reported that the AI-generated note functioned as a valuable record of the discussion, helping them verify checklist completion, promoting reflective conversations about communication, and enabling them to better understand the limitations of AI-assisted tools. These observations align with prior work suggesting that documentation of verbal discussions can enhance reflective learning and promote deliberate practice in team communication [31,32]. These findings also align with prior research showing that new technologies can be perceived as equally usable as established tools once users recognize their educational and workflow benefits [33].

Because learners were medical students and first-year interns with limited semantic understanding or familiarity with complex anesthesia and surgical terminology, they adhered closely to the scripted simulation scenarios. As a result, their handoffs were generally comprehensive, creating a ceiling effect on traditional performance measures such as completeness and limiting our ability to assess whether the ambient AI handoff assistant improved completeness. However, qualitative feedback indicated that learners valued the opportunity to practice structured team-based communication in a simulated setting and to engage with speech-to-text technology. This experience prompted broader discussions about communication strategies in high-stakes scenarios, focusing on the importance of closed-loop communication, clear and confident speech, and shared situational awareness to ensure effective information transfer. Further, interacting with imperfect AI output proved instructive, as learners observed transcription and checklist-mapping errors firsthand. In one instance, a learner with a foreign accent observed that her articulation of medical terminology was frequently inaccurately transcribed by the ambient AI handoff assistant, prompting a discussion about biases in training data and the limitations of speech-to-text systems. Trainees discussed the importance of vigilance of stereotyped assumptions and biased outputs with AI-generated content. Further, the limitations of the ambient AI handoff assistant in recognizing complex perioperative medical terminology also led to transcription and checklist mapping errors. Learners felt these inaccuracies became a productive feature of the simulation, enabling them to calibrate their trust in ambient AI outputs and also to understand how these technologies are assistive but still require human oversight. This led to discussion on automation complacency and its implications for patient safety with the use of AI technologies, prompting learner participants to reflect on the importance of verifying AI outputs [34,35] in future AI-assisted clinical environments. This fostered critical appraisal skills and transformed the AI assistant from a passive documentation tool into an active learning aid that promoted vigilance, AI literacy, and reflective communication. Future iterations of AI-assisted handoff training could vary the fidelity of AI output to examine how learners calibrate trust and maintain situational awareness when interacting with interactive systems.

Although this project primarily examined how ambient AI could support handoff education through automated documentation and verbal checklist workflow capture, it also demonstrated that high-stakes, multi-speaker handoff discussions can be structured into clinically and pedagogically meaningful documentation. The ambient AI handoff assistant was designed to map spoken communication to predefined checklist elements, thereby supporting the Educator during handoff simulation sessions and allowing greater focus on the Learners rather than on checklist reconciliation. During the error analysis phase, we assessed the ambient AI handoff assistant’s ability to map what was discussed to its corresponding checklist item. Most errors were attributed to difficulties in recognizing medical terminology and variability in speaker articulation. This finding was expected, given the inherent limitations of the Gemini 2.5 Flash Live API on which the system relies. Further, there were more transcription errors with the learners in simulation compared with the prior error analysis, likely reflecting the greater acoustic variability of real learning environments. Together, these factors contributed to downstream checklist mapping errors that resulted in imperfect checklist completeness assessment functionality of the ambient AI handoff assistant. Importantly, the goal of developing this system was to create an educational platform that would enable some transcription feedback of a high-stakes discussion, exposure to ambient AI, and assessment of whether AI could map spoken team communication to handoff checklist items as a feedback tool. Both educators and learners reflected on whether ambient AI technologies could play a role in real-world handoffs and used the simulation experience as a basis for discussing whether commercial ambient AI scribes might provide value in real-world OR-to-ICU handoff clinical workflows.

### Simulation as Essential Infrastructure for AI Evaluation in Health Care

Our work highlights the importance of simulation-based testing as essential infrastructure for preparing the medical workforce to engage safely with AI technologies [36]. Simulation provided a safe environment for experimentation, where medical trainees experienced an AI-assisted handoff workflow firsthand. It also allowed them to gain exposure to the limitations of AI technologies without exposing patients to risk. The findings also informed educators, prompting consideration of how AI assistance could be integrated into other educational modules. Simulation made it possible to observe behavioral and human-factor dynamics, such as how trainees modified their language when they knew a recording technology was in use. Further, it allowed Handoff QI leaders to consider whether to trial commercial ambient AI scribes in real-world handoff workflows and whether such technologies may play supportive roles in these workflows. These findings align with emerging frameworks for AI safety evaluation that emphasize controlled, risk-free environments where failure modes and sociotechnical evaluation can be systematically explored [37].

### Clinician-Led Innovation as a Model for Health Care AI Development

This study demonstrates that clinician-led development of AI technologies can produce viable AI proof-of-concept platforms for medical education purposes. This approach contrasts with traditional technology-driven innovation models where external developers create solutions that may not align with educational needs. The participatory design methodology [38,39] used in our co-design interviews exemplifies how domain expertise can be integrated into AI system development for real-world educational needs. In this project, clinicians designed and developed the ambient AI handoff assistant, which enabled them to gain a more realistic understanding of the AI’s capabilities and limitations. Furthermore, our experience suggests that clinicians can effectively translate clinical workflows into technical requirements. As an example, the development of the OR-to-ICU handoff note template using prompt engineering in Figure 3 [28], where items in the discussion are mapped to the checklist, demonstrates how clinical expertise can guide AI system design to produce outputs that are clinically meaningful.

### Limitations and Future Directions

This study was limited by its exploratory design, small sample size, and simulated environment. Novelty effects and learning curves were evident, as participants included trainees with varying degrees of medical experience and almost no exposure to the OR-to-ICU handoff process at UCLA. The single-site design and use of UCLA’s handoff checklist may also limit generalizability to other institutions with differing handoff checklist structures. However, the methodological framework developed in this study was designed to be adaptable across institutional handoff formats. Further, the error analysis was limited in scope and designed to assess feasibility rather than comprehensively evaluate model performance. Transcription errors that are specific to this ambient AI handoff assistant may not generalize to commercial ambient AI scribe platforms, which differ in accuracy, editing workflows, and user interaction. These differences may influence learners’ perceptions of ambient AI, particularly prior to direct exposure to commercial ambient AI scribe systems, and give them an inaccurate impression of how real-world commercial ambient AI scribe technologies work. Additionally, this analysis focused on checklist completeness and did not assess handoff sequence, which may affect comprehension and retention. Completeness alone may not reflect handoff quality; future studies should evaluate whether quantitatively more complete handoffs improve knowledge acquisition and retention among receiving clinicians. Additionally, further evaluation in larger samples and among more experienced trainee populations is needed to assess generalizability, usability, and effectiveness of the handoff assistant in supporting handoff education. Despite these limitations, simulation demonstrated the feasibility of using ambient AI technology to train learners in adhering to institutional checklists, reflecting on their communication practices, and gaining exposure to ambient AI technologies.

### Conclusions

This study highlights the potential of an ambient AI handoff assistant to enhance handoff education by converting real-time team dialogue into structured documentation for reflection and feedback. Through participatory co-design and simulation-based evaluation, we identified design requirements for integrating AI into handoff training that preserve authentic team-based communication and enrich reflective learning. The ambient AI handoff assistant demonstrated that complex team-based discussions can be mapped to real-world checklists. Although scripted simulations limited the ability to assess the handoff assistant’s impact on promoting improved checklist completeness, the AI-generated note emerged as a powerful learning tool that prompted critical discussion on communication clarity, calibration of trust in AI, and the boundaries of AI automation in human-AI collaboration. Imperfect AI output from the ambient AI handoff assistant encouraged learners to verify and question AI-generated information, fostering critical skills required for the safe use of AI in clinical settings. Taken together, these findings underscore how ambient AI technologies can support reflective, team-based learning while laying the groundwork for interactive AI systems that enhance safety, communication, and education in future clinical workflows.

## Supplementary material

10.2196/85666 Multimedia Appendix 1 Needs assessment and co-design interview script with clinician educators.

10.2196/85666 Multimedia Appendix 2 Representative prompt examples for the ambient AI assistant.

10.2196/85666 Multimedia Appendix 3 Debrief interview guide for simulation learner participants who used the ambient AI handoff assistant.

10.2196/85666 Multimedia Appendix 4 Final codebook for educator co-design and learner simulation sessions on the use of the AI in handoff education.

10.2196/85666 Checklist 1 COREQ checklist.

## Abbreviations

AI: artificial intelligence
API: application programming interface
COREQ: Consolidated Criteria for Reporting Qualitative Research
ICU: intensive care unit
LLM: large language model
OR: operating room
PTL: Physician Task Load Index
SUS: System Usability Scale
UCLA: University of California, Los Angeles
