# Supplementary material for: Exploring Ambient Artificial Intelligence to Enhance Learning and Feedback During Operating Room-to-Intensive Care Unit Handoffs: Co-Design and Simulation Study
Source: JMIR Med Educ. 2026 Jul 2;12:e85666. doi: 10.2196/85666 (PMC13326726; doi:10.2196/85666)
Supplement: Multimedia Appendix 1 [file mededu-v12-e85666-s001.docx]

| **Appendix 1. Needs Assessment and Co-Design Interview Script with Clinician Educators.** |
| --- |
| **Introductory Language:**  We are exploring how ambient AI handoff assistants, tools that passively listen to team conversations and generate structured documentation of the discussion can be used to enhance teaching and learning during operating room (OR) to intensive care unit (ICU) handoffs. Our work examines how exposure to these technologies in simulation-based education may help learners practice structured communication, apply handoff checklists more effectively, and reflect on their performance.  The goal of this project is to understand how these tools can support the education of medical students and residents in developing communication skills that improve the accuracy, consistency, and timeliness of information transfer during transitions of care. We also aim to explore how these tools influence interprofessional collaboration, learner engagement, and perceptions of handoff quality and safety within authentic educational settings.  This interview is being audio recorded to allow us to create a written transcript for later data analysis. When we transcribe the interview, your name and identifying information will not be included. You may choose not to answer any question at any time.  Before we begin, I’ll show you a demonstration of how an ambient AI handoff assistant captures and summarizes clinical conversations in real time and how it might surface relevant prompts or structured fields for documentation. [*demonstrate ambient AI handoff assistant use prior to start of interview*]  **Is it okay to proceed?** |
| Needs Assessment and Co-Design Session Facilitation Guide  Topic: OR-to-ICU Handoff Education Duration: 45 minutes |
| **1. Welcome & Grounding (5 minutes)**   - Facilitator script:   - Introduce the project purpose (use your Introductory Language).   - Emphasize: voluntary participation, confidentiality, anonymized transcripts.   - Explain the agenda: explore current handoff education challenges, imagine how AI might help, and co-create design requirements.   **2. Understanding Current Workflow and Current Handoff Training Educational Modules (20 minutes)**  Activity: Contextual Inquiry   - Prompt clinicians to “walk through a typical OR-to-ICU handoff.” - Probing questions:   - Where do breakdowns happen?   - What workarounds do you use?   - What makes a “good” handoff vs. a “bad” one? - Prompt clinicians to “reflect on existing handoff training.” - Probing questions:   - What aspects of existing handoff education are most effective?   - What challenges or gaps do you see in current handoff education for trainees?   - How do you provide feedback to learners on their handoff performance?   - In what ways could AI technology or simulation better support these learning objectives?   **3. Exploring AI Opportunities (20 minutes)**  Scenario Simulation   - Present a short case - Run through the handoff with/without AI support. - Ask:   - Where is this tool helpful? Distracting?   - What else should it do/not do?   **4. Reflection on Risks & Ethics (15 minutes)**  Discussion prompts:   - What worries you about this technology? - Could it create dependency, reduce vigilance, or harm communication? - What safeguards should be in place? - How might it affect learner education?   **5. Wrap-Up & Next Steps (5–10 minutes)**   - Summarize themes back to participants. - Share how their input will shape design requirements. - Offer ways to stay engaged (follow-up workshops, prototype testing). - Thank them for their time. |
