# Supplementary material for: Exploring Ambient Artificial Intelligence to Enhance Learning and Feedback During Operating Room-to-Intensive Care Unit Handoffs: Co-Design and Simulation Study
Source: JMIR Med Educ. 2026 Jul 2;12:e85666. doi: 10.2196/85666 (PMC13326726; doi:10.2196/85666)
Supplement: Multimedia Appendix 2 [file mededu-v12-e85666-s002.docx]

**Appendix 2.** Representative Prompt Examples for the Ambient AI Assistant.

/**

* @license

* Copyright 2025 Google LLC

* SPDX-License-Identifier: Apache-2.0

*/

import { GoogleGenAI, Type } from '@google/genai';

// --- SPEECH RECOGNITION AND SYNTHESIS SETUP ---

// Fix: Cast window to `any` to access non-standard SpeechRecognition APIs which are not in default TS types.

const SpeechRecognition =

(window as any).SpeechRecognition || (window as any).webkitSpeechRecognition;

const synthesis = window.speechSynthesis;

let recognition: any;

let recognitionRetryCount = 0;

const MAX_RETRIES = 3;

if (SpeechRecognition) {

recognition = new SpeechRecognition();

recognition.continuous = true;

recognition.interimResults = true;

}

// --- STATE MANAGEMENT ---

type AppState =

| { name: 'idle' }

| { name: 'listening'; transcript: string; startTime: number }

| { name: 'processing' }

| { name: 'verifying'; handoffNote: string; qualityMetrics: any }

| { name: 'metrics'; qualityMetrics: any, submissionError?: string }

| { name: 'summary'; analysis: ChecklistAnalysisResult, submissionError?: string }

| { name: 'error'; message: string };

interface ChecklistAnalysisResult {

completedItems: number;

totalItems: number;

missedItemsByCategory: Record<string, string[]>;

}

let state: AppState = { name: 'idle' };

let ai: GoogleGenAI | null = null;

let targetVoice: SpeechSynthesisVoice | null = null;

let committedTranscript = ''; // Persists transcript across recognition restarts

const root = document.getElementById('root')!;

// Define the exact labels and order for the Quality Metrics display

const metricsLabels: Record<string, string> = {

anesthesiologistName: 'Anesthesiologist Name',

surgeonName: 'Surgeon Name',

icuTeamRepresentativeName: 'ICU Team Representative Name',

icuNurseName: 'ICU Nurse Name',

pacuAttendingName: 'PACU Attending Name (if applicable)',

dateOfHandoff: 'Date of Handoff',

handoffDuration: 'Handoff Duration',

patientMrn: 'Patient MRN',

hospital: 'Hospital',

icu: 'ICU',

};

// This structure defines the master checklist for analysis.

// Labels must correspond to the labels in the handoff template.

const checklistStructure: Record<string, string[]> = {

"Introductions": [

"Anesthesia:", "Surgery:", "ICU Nurse:", "ICU Team:", "PACU Attending (if applicable):"

],

"Surgical Report": [

"Relevant medical history:", "Surgical course:", "Hemodynamic parameters:", "Any specific MAP/SBP/ICP goals?:", "Any specific fluid goals?:", "Any transfusion triggers?:", "Tubes/Lines/Drains:", "Dressings and/or wound care:", "Diet:", "VTE prophylaxis and/or anticoagulants:", "Other medications (e.g. immunosuppressants):", "Mobility (i.e. weight bearing restrictions):", "Family update: primary contact and last update:", "Length of stay: projected ICU/hospital course:", "Contact information and call triggers:"

],

"Anesthesia Report": [

"Significant medical history:", "Allergies/code/status/weight:", "Airway concerns:", "Last paralytic and reversal:", "Antibiotics:", "Pain medications:", "Other (pressors, anticoagulants, steroids, anti-emetics):", "Fluids:", "Blood products:", "Estimated blood loss:", "Uurine output:", "Last ABG:", "Last hemoglobin:", "Last blood glucose:", "Any placement challenges?:", "Neurologic status: baseline status?:", "Pain/sedation management:", "Respiratory status: vent settings:", "Hemodynamic stability:", "Current infusions: verify with source:", "Blood product availability:", "Medications given during transport?:", "Contact information:"

],

"Critical Care Team": [

"Open discussion with questions and/or clarifications?:", "Any unique patient details (e.g. pathways/research protocols)?:", "“Readback”: summarize immediate plan of care, prioritize clinical concerns and management plans:", "Confirm contact information and call triggers:", "Confirm ICU orders are placed:"

]

};

// --- METRICS TRACKING ---

let metricsData: any = {};

let handoffStartTime = 0;

let listeningStartTime = 0;

let verificationStartTime = 0;

let metricsVerificationStartTime = 0;

function setState(newState: AppState) {

state = newState;

render();

}

// --- VOICE MANAGEMENT ---

function initializeSpeechSynthesis() {

function findAndSetVoice() {

const voices = synthesis.getVoices();

// Prioritize the specific "Google UK English Female" voice.

targetVoice =

voices.find(

(voice) =>

voice.name === 'Google UK English Female' && voice.lang === 'en-GB'

) || null;

// Fallback 1: If the specific voice isn't found, find the first available generic en-GB voice.

if (!targetVoice) {

targetVoice = voices.find((voice) => voice.lang === 'en-GB') || null;

}

// Fallback 2: If no en-GB voice is found, use the first available English voice.

if (!targetVoice) {

targetVoice = voices.find((voice) => voice.lang.startsWith('en-')) || null;

}

}

// The 'voiceschanged' event is the reliable way to get the list of voices.

if (synthesis.onvoiceschanged !== undefined) {

synthesis.onvoiceschanged = findAndSetVoice;

}

findAndSetVoice(); // Call it once in case the voices are already loaded.

}

// --- API & PROMPT LOGIC ---

function initializeAi() {

if (!ai) {

ai = new GoogleGenAI({ apiKey: process.env.API_KEY });

}

}

async function generateReports(transcript: string, duration: string) {

if (!ai) throw new Error('AI not initialized');

const now = new Date();

const currentDate = now.toLocaleDateString();

const currentTime = now.toLocaleTimeString();

// Combine the date and time for the quality metrics to match the note.

const dateTimeForMetrics = `${currentDate}, ${currentTime}`;

// This template must be followed EXACTLY by the model.

const handoffTemplate = `

Date:

Time:

Introductions:

- Anesthesia:

- Surgery:

- ICU Nurse:

- ICU Team:

- PACU Attending (if applicable):

Surgical Report:

- Relevant medical history:

- Surgical course:

- Post-op Management Plan:

- Hemodynamic parameters:

Any specific MAP/SBP/ICP goals?:

Any specific fluid goals?:

Any transfusion triggers?:

- Tubes/Lines/Drains:

- Dressings and/or wound care:

- Diet:

- VTE prophylaxis and/or anticoagulants:

- Other medications (e.g. immunosuppressants):

- Mobility (i.e. weight bearing restrictions):

- Family update: primary contact and last update:

- Length of stay: projected ICU/hospital course:

- Contact information and call triggers:

Anesthesia Report:

- Significant medical history:

- Allergies/code/status/weight:

- Anesthetic course:

- Airway concerns:

- Medications:

Last paralytic and reversal:

Antibiotics:

Pain medications:

Other (pressors, anticoagulants, steroids, anti-emetics):

- Input:

- Fluids:

- Blood products:

- Output:

- Estimated blood loss:

- Urine output:

- Labs (if applicable):

- Last ABG:

- Last hemoglobin:

- Last blood glucose:

- Lines and access:

- Any placement challenges?:

- Current state and anticipatory guidance:

- Neurologic status: baseline status?:

- Pain/sedation management:

- Respiratory status: vent settings:

- Hemodynamic stability:

- Current infusions: verify with source:

- Blood product availability:

- Medications given during transport?:

- Contact information:

Critical Care Team:

- Open discussion with questions and/or clarifications:

- Any unique patient details (e.g. pathways/research protocols)?:

- “Readback”: summarize immediate plan of care, prioritize clinical concerns and management plans:

- Confirm contact information and call triggers:

- Confirm ICU orders are placed:

Handoff Duration:`;

const prompt = `I have this OR-to-ICU handoff transcript. Please extract the key details and use them to populate the following note template.

**TRANSCRIPT:**

---

${transcript}

---

**GOAL & INSTRUCTIONS:**

Your goal is to produce a clean, ready-to-use clinical note and a separate quality metrics report.

1. **Populate the Template:** Organize information from the transcript under the correct headings in the \`Handoff Note\` template below.

2. **Be Exact:** Preserve the exact headings, indentation, and structure of the template.

3. **Be Factual:** Use only information that is explicitly stated in the transcript.

4. **Handle Missing Info:** If a field is not addressed in the transcript, you **must** mark it as 'N/A'. Do not leave any fields blank.

5. **Handle Conflicting Info:** If conflicting information is given, use the most recent information provided and add \`(updated)\` next to it.

6. **Use Current Date/Time:**

- For the "Date" field in the note, use: ${currentDate}.

- For the "Time" field in the note, use: ${currentTime}.

- For the \`dateOfHandoff\` field in the \`qualityMetrics\` object, you **must** use this exact date and time string: "${dateTimeForMetrics}".

7. **Set Duration:** The "Handoff Duration" must be set to "${duration}".

8. **Format Output:** Produce a single, valid JSON object with two keys: \`handoffNote\` (containing the completed template as a multiline string) and \`qualityMetrics\` (containing the extracted metrics as a nested JSON object).

**HANDOFF NOTE TEMPLATE:**

---

${handoffTemplate}

---`;

const responseSchema = {

type: Type.OBJECT,

properties: {

handoffNote: {

type: Type.STRING,

description:

'A formatted string containing the full handoff note based on the template.',

},

qualityMetrics: {

type: Type.OBJECT,

properties: {

anesthesiologistName: { type: Type.STRING },

surgeonName: { type: Type.STRING },

icuTeamRepresentativeName: { type: Type.STRING },

icuNurseName: { type: Type.STRING },

pacuAttendingName: { type: Type.STRING },

dateOfHandoff: { type: Type.STRING },

handoffDuration: { type: Type.STRING },

patientMrn: { type: Type.STRING },

hospital: { type: Type.STRING },

icu: { type: Type.STRING },

},

},

},

required: ['handoffNote', 'qualityMetrics'],

};

try {

const response = await ai.models.generateContent({

model: 'gemini-2.5-flash',

contents: prompt,

config: {

responseMimeType: 'application/json',

responseSchema,

},

});

// The response is a string that needs to be parsed into JSON

const jsonString = response.text.trim();

const result = JSON.parse(jsonString);

if (result.handoffNote && result.qualityMetrics) {

return result;

} else {

throw new Error('Invalid response structure from AI.');

}

} catch (error) {

console.error('Error generating reports:', error);

throw new Error(

`Failed to process the transcript. Please try again. Details: ${error.message}`

);

}

}

// --- HELPER FUNCTIONS ---

function speak(text: string, onEnd?: () => void) {

try {

synthesis.cancel(); // Clear the queue

const utterance = new SpeechSynthesisUtterance(text);

if (targetVoice) { // Use the pre-selected target voice

utterance.voice = targetVoice;

}

utterance.onend = onEnd;

synthesis.speak(utterance);

} catch (error) {

console.error('Speech synthesis failed:', error);

}

}

function startListening() {

if (!recognition) {

setState({

name: 'error',

message: 'Speech recognition is not supported in this browser.',

});

return;

}

const startTime = Date.now();

setState({ name: 'listening', transcript: committedTranscript, startTime });

let sessionFinalTranscript = '';

recognition.onstart = () => {

// Successfully started, reset retry counter.

recognitionRetryCount = 0;

console.log('Speech recognition started.');

};

// Fix: Use `any` for the event type as SpeechRecognitionEvent is not a standard type in TypeScript.

recognition.onresult = (event: any) => {

let interimTranscript = '';

let currentSessionFinal = ''; // Rebuild from results for the current session

for (let i = 0; i < event.results.length; ++i) {

if (event.results[i].isFinal) {

currentSessionFinal += event.results[i][0].transcript;

} else {

interimTranscript += event.results[i][0].transcript;

}

}

sessionFinalTranscript = currentSessionFinal;

// Combine transcript from previous sessions with the latest from the current session

const fullTranscript = (committedTranscript + sessionFinalTranscript + ' ' + interimTranscript).trim();

// Update the state with the latest transcript. This is the single source of truth.

if (state.name === 'listening') {

setState({ ...state, transcript: fullTranscript });

}

if (

sessionFinalTranscript.toLowerCase().includes('generate note')

) {

handleGenerateNote();

}

};

// Fix: Use `any` for the event type as SpeechRecognitionErrorEvent is not a standard type in TypeScript.

recognition.onerror = (event: any) => {

console.warn(`Speech recognition error: ${event.error}. Retry count: ${recognitionRetryCount}`);

recognitionRetryCount++;

if (recognitionRetryCount > MAX_RETRIES) {

setState({ name: 'error', message: `Speech recognition failed after multiple retries. Please check your network connection and microphone permissions.` });

}

// The onend event will fire next, which will attempt a restart.

};

recognition.onend = () => {

console.log('Speech recognition ended.');

// Before restarting, "commit" the final transcript from the session that just ended.

if (sessionFinalTranscript) {

committedTranscript += sessionFinalTranscript;

}

// Automatically restart recognition if it stops (due to timeout or recoverable error),

// but only if we are still in the 'listening' state.

if (state.name === 'listening') {

console.log('Attempting to restart speech recognition...');

try {

recognition.start();

} catch (e) {

console.error('Failed to restart recognition:', e);

// If start() itself throws an error, it's likely a fatal issue.

if (recognitionRetryCount <= MAX_RETRIES) { // Prevent infinite loops

recognitionRetryCount++;

recognition.onend(); // Try again after a short delay might be better, but for now, direct retry.

} else {

setState({ name: 'error', message: 'Could not restart speech recognition. Please start over.' });

}

}

}

};

recognition.start();

}

function stopListening() {

if (recognition) {

recognition.stop();

}

}

function formatDuration(startTime: number, endTime: number): string {

const durationMs = endTime - startTime;

const seconds = Math.floor((durationMs / 1000) % 60);

const minutes = Math.floor(durationMs / (1000 * 60));

return `${minutes}m ${seconds}s`;

}

function formatDurationMsToSeconds(durationMs: number): number {

return parseFloat((durationMs / 1000).toFixed(2)); // Return seconds with 2 decimal places

}

/**

* Parses a handoff note string and returns an HTML string with labels bolded.

* @param noteString The raw string of the handoff note.

* @returns An HTML string with labels wrapped in <strong> tags.

*/

function formatHandoffNoteForDisplay(noteString: string): string {

const lines = noteString.split('\n');

const formattedLines = lines.map(line => {

// A regex to find the label part of a line (up to and including the colon).

const match = line.match(/^([^:]+:\s*)/);

if (match) {

const label = match[1];

const value = line.substring(label.length);

// Basic HTML escaping for the value to prevent accidental HTML injection

const escapedValue = value.replace(/</g, '&lt;').replace(/>/g, '&gt;');

return `<strong>${label}</strong>${escapedValue}`;

}

// If no colon is found, return the line as is.

return line;

});

return formattedLines.join('<br>');

}

/**

* Analyzes the final handoff note against the master checklist to determine completeness.

* @param noteText The final, verified handoff note text.

* @returns An object with counts of completed/total items and a breakdown of missed items.

*/

function analyzeChecklistCompleteness(noteText: string): ChecklistAnalysisResult {

const allItems = Object.values(checklistStructure).flat();

const totalItems = allItems.length;

const completedSet = new Set<string>();

const lines = noteText.split('\n');

lines.forEach(line => {

const trimmedLine = line.trim();

const colonIndex = trimmedLine.indexOf(':');

if (colonIndex > -1) {

const label = trimmedLine.substring(0, colonIndex + 1);

const value = trimmedLine.substring(colonIndex + 1).trim();

if (value !== '' && value.toLowerCase() !== 'n/a') {

// Find which master checklist item this label corresponds to.

// Use .endsWith() for a more specific match than .includes()

const matchingItem = allItems.find(item => label.trim().endsWith(item));

if (matchingItem) {

completedSet.add(matchingItem);

}

}

}

});

const completedItems = completedSet.size;

const missedItemsByCategory: Record<string, string[]> = {};

for (const category in checklistStructure) {

missedItemsByCategory[category] = [];

checklistStructure[category].forEach(item => {

if (!completedSet.has(item)) {

// Remove trailing colon for cleaner display

missedItemsByCategory[category].push(item.replace(/:$/, ''));

}

});

if (missedItemsByCategory[category].length === 0) {

delete missedItemsByCategory[category];

}

}

return { completedItems, totalItems, missedItemsByCategory };

}

async function submitMetrics() {

// Flatten the quality metrics into the main object

const payload = {

...metricsData,

...metricsData.qualityMetrics,

};

// We don't need the nested object anymore

delete payload.qualityMetrics;

const formData = new URLSearchParams();

for (const key in payload) {

// Ensure value is a string, as URLSearchParams requires

formData.append(key, String(payload[key]));

}

// DEBUGGING: Log the payload to the browser's developer console

console.log("Submitting the following data to Google Sheet:", payload);

await fetch(SPREADSHEET_URL, {

method: 'POST',

mode: 'no-cors', // Important for simple Google Apps Script POST requests

body: formData, // The browser will automatically set the Content-Type to application/x-www-form-urlencoded

});

// Note: With 'no-cors', we can't read the response, so this is fire-and-forget.

// We assume success if fetch doesn't throw a network error.

}

// --- EVENT HANDLERS ---

function handleStartHandoff() {

initializeAi();

// Reset and start metrics capture

metricsData = {};

metricsData.submissionTimestamp = new Date().toISOString();

handoffStartTime = Date.now();

listeningStartTime = Date.now();

recognitionRetryCount = 0; // Reset retry counter for new session

committedTranscript = ''; // Reset for the new handoff session

speak('Welcome Handoff Team!', () => {

startListening();

});

}

async function handleGenerateNote() {

if (state.name !== 'listening') return;

stopListening(); // Stop recognition first to finalize the last utterance

const { transcript, startTime } = state; // Now reliably get the transcript from state

const duration = formatDuration(startTime, Date.now());

// Capture listening metrics

const listeningEndTime = Date.now();

metricsData.listeningTime = formatDurationMsToSeconds(listeningEndTime - listeningStartTime);

metricsData.originalTranscript = transcript;

setState({ name: 'processing' });

try {

const reports = await generateReports(transcript, duration);

// Capture AI-related metrics

metricsData.aiGeneratedNote = reports.handoffNote;

metricsData.qualityMetrics = reports.qualityMetrics;

verificationStartTime = Date.now(); // Start verification timer

setState({

name: 'verifying',

handoffNote: reports.handoffNote,

qualityMetrics: reports.qualityMetrics,

});

speak('Please review the note for verification.');

} catch (error) {

setState({ name: 'error', message: (error as Error).message });

}

}

function handleVerification() {

if (state.name !== 'verifying') return;

// Capture verification metrics

const verificationEndTime = Date.now();

metricsData.noteVerificationTime = formatDurationMsToSeconds(verificationEndTime - verificationStartTime);

// Capture the latest edits from the contenteditable div.

const noteDisplay = document.getElementById('note-display') as HTMLDivElement;

metricsData.finalVerifiedNote = noteDisplay.innerText;

metricsVerificationStartTime = Date.now(); // Start metrics review timer

setState({ name: 'metrics', qualityMetrics: state.qualityMetrics });

speak('Here are the Quality Metrics.');

}

function handleStartNew() {

setState({ name: 'idle' });

}

function handleProceedToSummary() {

if (state.name !== 'metrics') return;

// Start with a copy of the current metrics

let updatedMetrics = { ...state.qualityMetrics };

// Capture edited metrics from the contenteditable div.

const metricsDisplay = document.getElementById('metrics-display') as HTMLDivElement;

if (metricsDisplay) {

const editedText = metricsDisplay.innerText;

const lines = editedText.split('\n');

const labelsToKeys = Object.entries(metricsLabels).reduce((acc, [key, label]) => {

acc[label] = key;

return acc;

}, {} as Record<string, string>);

lines.forEach(line => {

const colonIndex = line.indexOf(':');

if (colonIndex > -1) {

const label = line.substring(0, colonIndex).trim();

const value = line.substring(colonIndex + 1).trim();

const key = labelsToKeys[label] as keyof typeof updatedMetrics;

if (key) {

updatedMetrics[key] = value;

}

}

});

}

// Update the global metrics object for submission

metricsData.qualityMetrics = updatedMetrics;

// Capture final timings for metrics page

const metricsVerificationEndTime = Date.now();

metricsData.qualityMetricsVerificationTime = formatDurationMsToSeconds(metricsVerificationEndTime - metricsVerificationStartTime);

// Analyze the verified note for completeness

const analysis = analyzeChecklistCompleteness(metricsData.finalVerifiedNote);

setState({ name: 'summary', analysis });

}

async function handleSubmitAndStartNew() {

if (state.name !== 'summary') return;

const handoffEndTime = Date.now();

metricsData.totalHandoffDuration = formatDurationMsToSeconds(handoffEndTime - handoffStartTime);

// Update UI to show submission in progress

const button = document.getElementById('final-submit-btn') as HTMLButtonElement;

button.disabled = true;

button.textContent = 'Submitting...';

try {

await submitMetrics();

// On success, reset the app

handleStartNew();

} catch (error) {

console.error('Submission failed:', error);

// On failure, show an error and re-enable the button

setState({

...state,

submissionError: 'Submission failed. Please try again.',

});

}

}

// --- RENDER FUNCTIONS ---

function render() {

root.innerHTML = `

<header>

<h1>Ambient AI Handoff Assistant</h1>

</header>

<main id="main-content"></main>

`;

const main = document.getElementById('main-content')!;

switch (state.name) {

case 'idle':

main.innerHTML = `

<div class="idle-view">

<h2>An ambient AI assistant for OR-to-ICU handoffs.</h2>

<button id="start-btn">Start Handoff</button>

<img src="https://drive.google.com/thumbnail?id=1LYc36OhmmVxcUz0w-ZE_lv6YDEs7Aj7l&sz=w2048" alt="Partner Logo Left" class="idle-logo-left" />

<img src="https://drive.google.com/thumbnail?id=1uGXCdTzd2l_1Zg3g_PtOwzXj-I-IhFZq&sz=w2048" alt="Partner Logo" class="idle-logo" />

</div>

`;

document

.getElementById('start-btn')!

.addEventListener('click', handleStartHandoff);

break;

case 'listening':

main.innerHTML = `

<div class="listening-view">

<div class="checklist-container">

<!-- Image is now a background via CSS -->

</div>

<div class="transcript-container">

<h3>Live Transcript</h3>

<div class="status">${MIC_ICON_SVG} Listening...</div>

<div id="transcript"></div>

<div class="actions">

<button id="generate-btn">Generate Note</button>

</div>

</div>

</div>

`;

// The transcript is now rendered directly from the state

document.getElementById('transcript')!.innerText = state.transcript;

document

.getElementById('generate-btn')!

.addEventListener('click', handleGenerateNote);

break;

case 'processing':

main.innerHTML = `

<div class="processing-view">

<div class="loader"></div>

<h2>Generating Handoff Note...</h2>

<p>Analyzing transcript with AI. This may take a moment.</p>

</div>

`;

break;

case 'verifying':

const formattedNote = formatHandoffNoteForDisplay(state.handoffNote);

main.innerHTML = `

<div class="results-view">

<h3>Handoff Note (Editable)</h3>

<div id="note-display" class="note-display" contenteditable="true">${formattedNote}</div>

<div class="actions">

<button id="verify-btn" class="success">Verify & Proceed</button>

</div>

</div>

`;

document.getElementById('verify-btn')!.addEventListener('click', handleVerification);

break;

case 'metrics':

const orderedKeys = Object.keys(metricsLabels);

const qualityMetrics = state.qualityMetrics;

const formattedMetrics = orderedKeys.map(key => {

const label = metricsLabels[key as keyof typeof metricsLabels];

const value = qualityMetrics[key] ?? 'N/A';

// Basic HTML escaping for the value to prevent accidental HTML injection

const escapedValue = String(value).replace(/</g, '&lt;').replace(/>/g, '&gt;');

return `<strong>${label}:</strong> ${escapedValue}`;

}).join('<br>');

main.innerHTML = `

<div class="results-view">

<h3>Quality Metrics Extract (Editable)</h3>

<div id="metrics-display" class="metrics-display" contenteditable="true">${formattedMetrics}</div>

<div class="actions">

<button id="summary-btn">View Summary</button>

</div>

</div>

`;

document.getElementById('summary-btn')!.addEventListener('click', handleProceedToSummary);

break;

case 'summary':

const { completedItems, totalItems, missedItemsByCategory } = state.analysis;

const percentage = totalItems > 0 ? Math.round((completedItems / totalItems) * 100) : 100;

let missedItemsHtml = '';

const missedCategories = Object.keys(missedItemsByCategory);

if (missedCategories.length === 0) {

missedItemsHtml = `<p class="all-items-complete">Great job! All checklist items were discussed.</p>`;

} else {

missedItemsHtml = missedCategories.map(category => `

<div class="missed-category">

<h4>${category}</h4>

<ul>

${missedItemsByCategory[category].map(item => `<li>${item}</li>`).join('')}

</ul>

</div>

`).join('');

}

main.innerHTML = `

<div class="summary-view">

<div class="summary-quantitative">

<h3>Checklist Completeness</h3>

<div class="progress-circle" style="--p:${percentage};">

<div class="progress-percentage">${percentage}%</div>

</div>

<p class="summary-text">${completedItems} of ${totalItems} items discussed</p>

</div>

<div class="summary-qualitative">

<h3>Items Not Discussed</h3>

<div class="missed-items-list">

${missedItemsHtml}

</div>

</div>

</div>

<div class="actions">

<button id="final-submit-btn">Handoff Complete!</button>

${state.submissionError ? `<p class="submission-error">${state.submissionError}</p>` : ''}

</div>

`;

document.getElementById('final-submit-btn')!.addEventListener('click', handleSubmitAndStartNew);

break;

case 'error':

main.innerHTML = `

<div class="error-view">

<h2>An Error Occurred</h2>

<p style="color: var(--error-color);">${state.message}</p>

<button id="start-over-btn">Start Over</button>

</div>

`;

document.getElementById('start-over-btn')!.addEventListener('click', handleStartNew);

break;

}

}

// Initial Load

initializeSpeechSynthesis();

render();
