# Supplementary material for: Exploring Ambient Artificial Intelligence to Enhance Learning and Feedback During Operating Room-to-Intensive Care Unit Handoffs: Co-Design and Simulation Study
Source: JMIR Med Educ. 2026 Jul 2;12:e85666. doi: 10.2196/85666 (PMC13326726; doi:10.2196/85666)
Supplement: Multimedia Appendix 3 [file mededu-v12-e85666-s003.docx]

#

| **Appendix 3. Debrief Interview Guide for Simulation Learner Participants Who Used the Ambient AI Handoff Assistant** |
| --- |
| 1. **Prior experience**    1. Before today, what experience did you have with AI tools in your clinical or personal life? 2. **Understanding of the tool**    1. In your own words, how would you describe the purpose and functionality of the Handoff AI assistant during OR-to-ICU handoff training? 3. **Expectations**    1. What were your expectations going into this simulation about using the AI assistant for handoff communication training?    2. To what extent did your experience meet, exceed, or fall short of those expectations? 4. **Effect on handoff preparation & delivery**    1. Did you feel the need to adjust your communication style or the way you presented information because the AI assistant was active? 5. **Impact on documentation**    1. How would you describe the quality and usefulness of the handoff summary generated by the AI assistant?    2. Did the notes accurately capture what was said during the handoff? 6. **Impact on interprofessional communication**    1. In what ways, if any, did the AI assistant change the flow of interprofessional communication during the handoff?    2. Were there moments where you or others hesitated or withheld information because the assistant was recording? 7. **Effect on patient care**    1. What potential impacts—positive or negative—could AI scribe–generated documentation have on patient care during or after handoffs? 8. **Concerns about recording**    1. Can you think of situations where you might feel uncomfortable with an AI assistant recording the handoff conversation? 9. **Final reflections**    1. Looking back on this simulation, what is the most important takeaway you’d want to share about your experience with the Handoff AI assistant? |
