# Supplementary material for: Exploring Ambient Artificial Intelligence to Enhance Learning and Feedback During Operating Room-to-Intensive Care Unit Handoffs: Co-Design and Simulation Study
Source: JMIR Med Educ. 2026 Jul 2;12:e85666. doi: 10.2196/85666 (PMC13326726; doi:10.2196/85666)
Supplement: Multimedia Appendix 4 [file mededu-v12-e85666-s004.docx]

**Appendix 4. Final Codebook for Educator Co-Design and Learner Simulation Sessions on the Use of the AI in Handoff Education**

| **Code** | **Definition** |
| --- | --- |
| **Codebook A: Educator Co-Design Session Codebook** | |
| Promoting Accountability and Professional Communication | Awareness of AI recording or documentation encouraging more deliberate, mindful, and professional communication among trainees. |
| Enhancing Reflective Learning | AI-generated transcripts and summaries provide learners with opportunities to review their own communication and identify gaps, improve clarity, and strengthen handoff performance. |
| Exposure and Readiness for AI | Learners’ or educators’ experiences with AI tools in simulation or education, including curiosity, confidence, or uncertainty about AI’s accuracy or role and how it might prepare them for real-world use of AI in perioperative or ICU settings. |
| Calibration of Trust in AI Output | Exposure to AI mistakes is instructive for critically assessing and calibrating trust in AI-generated documentation. |
| Reducing Documentation Burden | References to AI automating or simplifying note-taking tasks. |
| Ethical and Privacy Considerations | Mentions of ethical and privacy issues related to AI use in training, including recording, data storage, consent, and impacts on psychological safety. |
| Transformation of Educator Role | AI may shift educators’ responsibilities from manual note-taking toward higher-order teaching roles, such as observation and formative feedback. |
| Validation of AI Output by Learners | Statements reflecting learners’ abilities on verifying the accuracy of AI-generated transcripts. |
| Validation of AI Output by Educators | Statements where educators emphasize the importance of reviewing and validating AI outputs before relying on them in assessment or teaching. |
| Standardizing Educational Language | Mentions of AI contributing to consistent terminology, structure, or phrasing in handoff communication to support shared mental models and understanding. |
| Clinical Workflow Integration | References to how AI technologies could be implemented in real workflows, including considerations of feasibility, safety, and coordination among perioperative and ICU teams. |
| AI Teaching Utility | Mentions of how AI-generated transcripts or summaries could be used by educators to guide debriefing, deliver structured feedback, or assess communication performance. |
| Psychological Safety | References to how awareness of AI recording may influence learners’ willingness to speak openly, ask questions, make mistakes during simulation or teaching sessions. |
| Authentic Participation | Mentions of how AI technologies might inhibit participation, self-censorship, or anxiety about being evaluated. |
| Perceived Accuracy of AI | Mentions of participants evaluating how accurate or reliable the AI assistant is in capturing or summarizing handoff discussions. |
| Freeing Educator Cognitive Load | Mentions of how AI may reduce educators cognitive load. |
| Handoff Assistant Design Needs | Any mention of design requirements for Ambient AI Handoff Assistant. |
|  | |
| **Codebook B. Learner Simulation Session Codebook** | |
| Exposure and Readiness for AI | Learners’ first experiences with AI assistants in a clinical simulation, including curiosity, excitement, or apprehension about accuracy and reliability. |
| Enhancing Reflective Learning | Use of AI-generated transcripts or summaries to review performance, identify missed information, and improve future communication. |
| Promoting Accountability and Professional Communication | Learners’ heightened mindfulness in speech and behavior when aware that AI is documenting their interactions. |
| Perceived Usefulness and Usability | Learners’ views on how intuitive, seamless, and relevant the AI tool felt within the simulation workflow. |
| Trust and Error Sensitivity | Reflections on AI mistakes, perceived fairness, and willingness to rely on AI outputs for feedback or documentation accuracy. |
| Ethical and Privacy Awareness | Learners’ comfort levels with being recorded, and their understanding of how transcripts are used or stored. |
| Reducing Cognitive Load | Learners’ sense that AI automation allowed them to focus more on communication and teamwork instead of remembering details. |
| Peer Interaction and Group Dynamics | Comments on how AI presence shaped communication among team members (e.g., speaking differently when being recorded, or adjusting roles). |
| Authenticity and Realism of Simulation | Perceptions of how the presence of the AI assistant affected the realism, flow, or authenticity of the simulation exercise. |
| Learning Curve and Adaptation | Learners’ reflections on the initial adjustment period to having an AI “listening,” and how comfort or confidence changed over time. |
| Feedback Integration | Learners’ appreciation for the AI’s potential to provide structured feedback or highlight communication gaps when reviewed with instructors. |
| Clinical Workflow Integration | Learners’ perspectives on how AI scribes might function in real clinical settings, including accuracy during handoffs, speed, and alignment with existing norms. |
| Emotional Response and Adaptation | Learners’ emotional reactions to the AI’s presence, including initial self-consciousness, discomfort, curiosity, or eventual normalization of being recorded. |
| Perceived Accuracy and Output Quality | Learners’ evaluations of how well the AI captured speech, medical terminology, and communication details, including perceptions of reliability, transcription errors, and overall quality of AI-generated content. |
