# Supplementary material for: Exploring Ambient Artificial Intelligence to Enhance Learning and Feedback During Operating Room-to-Intensive Care Unit Handoffs: Co-Design and Simulation Study
Source: JMIR Med Educ. 2026 Jul 2;12:e85666. doi: 10.2196/85666 (PMC13326726; doi:10.2196/85666)
Supplement: Checklist 1 [file mededu-v12-e85666-s005.pdf]

Appendix 5. COREQ Checklist.

Checklist Item	Guide Questions	Fulfilled or Addressed ?	Evidence/Comments
1. Interviewer/facilitator	Which author/s conducted the interview or focus group?	Yes	Stated in Methods section
2. Credentials	What was the researcher's credentials?	Yes	Stated in Methods section; also seen in author list on title page
3. Occupation	What was their occupation at the time of the study?	Yes	Stated in Methods section
4. Gender	Was the researcher male or female?	No	Not relevant to the methodology of this study
5. Experience and training	What experience or training did the researcher have?	Yes	Stated in Methods section
6. Relationship established	Was a relationship established prior to study commencement?	Yes	Stated in Methods section
7. Participant knowledge of the interviewer	What did the participants know about the researcher?	Yes	Stated in Methods section; obtained verbal informed consent, which stated research purpose
8. Interviewer characteristics	What characteristics were reported about the interviewer/facilitator?	Yes	Stated in Methods section
9. Methodological orientation and Theory	What methodological orientation was stated to underpin the study?	Yes	Stated in Methods section; grounded theory approach

10. Sampling	How were participants selected?	Yes	Stated in Methods, Interviews and Focus Groups section; based on learner availability and self-identification, so convenience
11. Method of approach	How were participants approached?	Yes	Stated in Methods, Study Population and Recruitment section; e-mails and announcements
12. Sample size	How many participants were in the study?	Yes	Stated in Results section
13. Non-participation	How many people refused to participate or dropped out? Reasons?	No	Learners self-identified to participate, so refusal is not relevant
14. Setting of data collection	Where was the data collected?	Yes	Stated in Methods section
15. Presence of non-participants	Was anyone else present besides the participants and researchers?	Yes	Stated in Methods section; nobody else present
16. Description of sample	What are the important characteristics of the sample?	Yes	Stated in Methods section
17. Interview guide	Were questions, prompts, guides provided by the authors? Was it pilot tested?	Yes	Interview script provided as Appendix 1 and 2
18. Repeat interviews	Were repeat interviews carried out? If yes, how many?	No	Stated in Methods section; subjects each participated in one interview or focus group
19. Audio/visual recording	Did the research use audio or visual recording to collect the data?	Yes	Stated in Methods section

20. Field notes	Were field notes made during and/or after the interview or focus group?	Yes	Stated in Methods section; field notes taken during interviews/focus groups
21. Duration	What was the duration of the interviews or focus group?	Yes	Stated in Results section
22. Data saturation	Was data saturation discussed?	Yes	Stated in Results section; reached by consensus of researchers
23. Transcripts returned	Were transcripts returned to participants for comment and/or correction?	Yes	Stated in Methods section
24. Number of data coders	How many data coders coded the data?	Yes	Stated in Methods section; two trained coders
25. Description of the coding tree	Did authors provide a description of the coding tree?	Yes	Codebook provided as Appendix 3
26. Derivation of themes	Were themes identified in advance or derived from the data?	Yes	Stated in Methods section; authors developed codebook by reading through data, themes subsequently derived
27. Software	What software, if applicable, was used to manage the data?	Yes	Stated in Methods section
28. Participation checking	Did participants provide feedback on the findings?	Yes	Stated in Methods section
29. Quotations presented	Were participant quotations presented to illustrate the themes/findings? Was each quotation identified?	Yes	Quotations, along with clinician subject indicators, provided in Results section

30. Data and findings consistent	Was there consistency between the data presented and the findings?	Yes	Data presented and described in Results are consistent with findings relayed in Results and Discussion
31. Clarity of major themes	Were major themes clearly presented in the findings?	Yes	Discussed in Results section and further described in Table 2 and 5
32. Clarity of minor themes	Is there a description of diverse cases or discussion of minor themes?	Yes	Discussed in Results section

30/32 Addressed; Remaining 2 deemed not relevant to this study
